# Fungal Super Glue: The Biofilm Matrix and Its Composition, Assembly, and Functions

**DOI:** 10.1371/journal.ppat.1005828

**Published:** 2016-09-29

**Authors:** Kaitlin F. Mitchell, Robert Zarnowski, David R. Andes

**Affiliations:** Departments of Medicine and Microbiology and Immunology, University of Wisconsin, Madison, Wisconsin, United States of America; Geisel School of Medicine at Dartmouth, UNITED STATES

## The Common Thread of Microbial Biofilms

**Fig 1:**
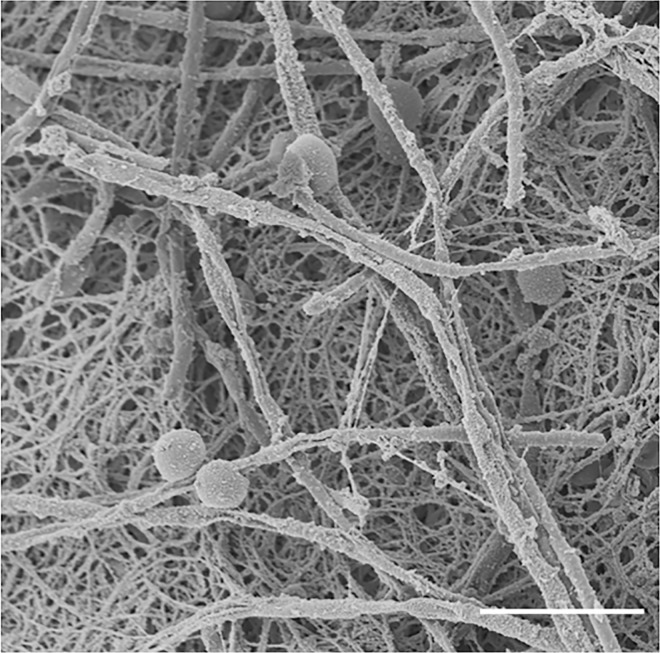
Scanning electron micrograph of a *Candida albicans* biofilm grown on a rat central venous catheter. The image demonstrates fungal yeast and hyphal cell morphologies as well as abundant extracellular matrix material. Scale bar represents 10 μm.

Biofilms are arguably the most common state of microbial growth found in nature and in patients infected with pathogenic organisms. A canonical feature of prokaryotic and eukaryotic biofilms is their production of an extracellular matrix (Fig 1). The matrix provides a protective environment for biofilm cells, offering a three-dimensional framework for both surface adhesion and cell cohesion [1,2]. In addition, this extracellular material controls cell dispersion from the biofilm and provides a nutrient source for the community [3]. The physical barrier formed by the matrix is also clinically relevant, as it shields cells from environmental threats, including immune cells and antimicrobial drugs used for treatment [4,5]. This defensive characteristic has been demonstrated for biofilms formed by diverse fungal pathogens, including *Aspergillus*, *Candida*, *Cryptococcus*, and *Saccharomyces*, with *Aspergillus fumigatus* and *Candida albicans* being the best studied [2,5–8]. Biofilm-associated *Candida* infections are the fourth cause for nosocomial infections (predominantly infecting medical devices), which may lead to systemic infection associated with high mortality rates. *Candida spp*. are also the most common cause of mucosal infection of the oral and vaginal sites, where biofilm infection has been increasingly recognized. Despite the ubiquitous nature of the biofilm matrix, we are only beginning to understand the synthesis and composition of this material for a handful of species. This review will discuss select components of the extracellular matrix of fungal biofilms, including their synthesis, structure, and function.

## A Complex Assembly of Self, Host, and Neighbor

Two themes are common in the composition of microbial matrix. First, there is a contribution from each of the four macromolecular classes: carbohydrate, protein, lipid, and nucleic acid (Table 1). Second, there are complex interactions among the matrix components. The abundance and specific chemistry of biofilm matrices among these components can be as diverse as the microbes that produce them, in addition to the conditions under which biofilms are formed. The greatest divergence in matrix composition appears at the protein level [9–11]; however, differences have also been observed in carbohydrate content and have been the focus of most studies. For example, *A*. *fumigatus*, *C*. *albicans*, *Cryptococcus neoformans*, and *Saccharomyces cerevisiae* have each been shown to produce distinct, complex matrix polysaccharides frequently composed of two or more monosaccharide components [11]. Interestingly, many of the matrix components are similar, at least in part, to cell wall constituents. In fact, certain cell wall production enzymes are important for the production of a matrix [12,13]. However, the mature matrix structures most often vary from their cell wall counterparts in size, branching, and sometimes in the combination of monosaccharide components. This suggests that matrix polysaccharides are either distinct from the cell wall or further modified after release from the cell wall. In the case of *C*. *albicans*, assembly of the mannan-glucan complex (MGCx) was found to occur in the extracellular space, demonstrating a critical role for the polysaccharide modification enzymes identified in the matrix proteome. There has been minimal structural study of matrix lipid composition and assembly; the observations demonstrated both similarities and differences between matrix components and the cell plasma membrane [11,14].

**Table 1:** Fungal Matrix Polysaccharide Content and Function.

Species	Characterized matrix components*	Drug resistance	Immune resistance	Relevant sources
*Aspergillus fumigatus*	Carbohydrates: GAG, galactomannan, α-1,3 glucan, monosaccharides (43%); proteins: major antigens and hydrophobins (40%); lipids (14%); melanin, polyols, eDNA	GAG, eDNA	GAG	[7,27,32,37]
*Candida albicans*	Glycoproteins and neutral polysaccharides (25%); 458 distinct proteins (55%); lipids, including neutral and polar glycerolipids and sphingolipids (15%); nucleic acids (5%); phosphorus, uronic acid, hexosamine	MGCx (α-mannan, β-1,6 glucan, and β-1,3 glucan), eDNA	β-1,3 glucan	[11,21,38]
*Cryptococcus neoformans*	Carbohydrates: glucurunoxylomannan, xylose, mannose, glucose, galactoxylomannan	—	—	[6]
*Saccharomyces cerevisiae*	Carbohydrates including glucose, mannose, and galactose; proteins in lower abundance including Tdh3, Hsp26, and Sod2	—	—	[8,39]

*Percent values in parentheses indicate relative abundance for certain components within the extracellular matrix. GAG: galactosaminogalactan; MCCx: mannan-glucan complex; eDNA: extracellular DNA.

Under in vivo conditions, the extracellular matrix complexity increases further. *C*. *albicans* biofilms in three common infection site models each produced a matrix that contained a striking amount of host components—up to 98% of matrix proteins were of host origin [15]. The most abundant host proteins varied based on the host niche: hemoglobin, albumin, and alpha globulins in the venous catheter model; amylase and hemoglobin in the denture model; and fibrinogen, keratin, and hemoglobin in the urinary catheter model. However, there was a conserved group of proteins that included the matricellular proteins fibrinogen, fibronectin, hemoglobin, and vitronectin, suggesting biofilm relevance across infection sites. DNA from the host is also a common matrix factor that has been linked to biofilm structural integrity and total matrix production by *Aspergillus* biofilms [16].

Biofilm infections are often polymicrobial, with combinations of bacterial–bacterial and bacterial–fungal species described [17,18]. The matrix composition of mixed-species biofilms has not been closely examined. However, recent study of a *C*. *albicans* and *Staphylococcus aureus* mixed biofilm found the *Candida-*derived extracellular matrix encased the bacterial community [19]. It is plausible that entities from each species might help or hinder the production of matrix by the other, for example, the complex 3D network of matrix-rich *Candida* biofilm may conceivably provide a hypoxic microenvironment that nourishes the growth of anaerobic bacteria [20]. Future experiments in this area will be tasked with identifying changes in the mixed biofilm matrix compared to either biofilm species alone.

## Key Components for a Protective Matrix

One of the medically relevant traits of the biofilm extracellular matrix is its ability to protect cells from extraordinarily high levels of anti-infectives. The initial link between *Candida* matrix and resistance was discovered by the Douglas laboratory when they correlated matrix abundance with biofilm tolerance to the antifungal drugs amphotericin B and fluconazole [21]. The *Candida* matrix polysaccharide first linked to biofilm resistance to multiple drugs was β-1,3 glucan [13,22,23]; through a mechanism of drug sequestration, this matrix polysaccharide prevents drugs from reaching their cellular targets. β-1,3 glucan has also been suggested to prevent neutrophil activation, accounting for *Candida* biofilm resistance to killing by these innate immune cells [24]. The exact nature of the matrix–antifungal drug interaction remains undefined. However, preliminary nuclear magnetic resonance (NMR) interaction studies and the differing physiochemical properties of the antifungal drugs impacted by this resistance mechanism suggest a noncovalent drug–matrix interaction [11]. While most matrix biochemical studies have been undertaken with *C*. *albicans*, phenotypic studies suggest that the matrix drug sequestration phenomenon is common for other *Candida* species [25].

More recent work found surprisingly low levels of β-1,3 glucan but found larger quantities of β-1,6 glucan and α-mannan, which interact to form an MGCx [11,26]. This polysaccharide interaction was discovered to be key for protection of the biofilm from drug treatment.

Compared to bacterial biofilms, where extracellular DNA (eDNA) is an established mode of horizontal gene transfer, *C*. *albicans* eDNA is largely noncoding [11]. Autolysis likely contributes to eDNA entering the matrix, as chitinase activity increases DNA release by *Aspergillus* in biofilms [16,27]. The exact mechanism of how eDNA might contribute to drug resistance remains unclear but may also be due to reduced drug penetration.

The role of matrix in multispecies interactions has not been intensively studied. However, it does appear that bacterial biofilm growth with *Candida* is linked to protection of the prokaryotes from antibacterial agents. For example, *S*. *aureus* grown in a mixed biofilm with *C*. *albicans* had heightened resistance to vancomycin [28]. Furthermore, recent work with an *S*. *aureus* and *Candida* mixed model identified a role for the *C*. *albicans* matrix mannan-glucan complex in antibacterial shielding [29]. *C*. *albicans* matrix carbohydrate was also found to provide protection to *Escherichia coli* against the antibiotic ofloxacin in a mixed biofilm scenario [30]. These findings further demonstrate the nonspecific nature of protection by the matrix from numerous drugs with widely divergent physiochemical properties. Interestingly, the galactosaminogalactan (GAG) matrix polysaccharide of *Aspergillus* has been similarly linked to antifungal protection [31]. Additionally, GAG affects virulence through the masking of cell wall β-1,3 glucan and modulating host immune responses, including neutrophil apoptosis [31–33].

## Unraveling the Matrix: Exploitation of Biofilm Drug Targets

During biofilm growth, production of extracellular matrix proceeds rapidly during the first 24 hours of maturation. Attempts to target this biofilm component suggest therapeutic promise for either the prevention or treatment of biofilm infections. It is intriguing that the most effective of the currently available antifungal drug classes for treatment of *Candida* biofilms are the echinocandin antifungals, which inhibit cell wall β-1,3 glucan synthesis [34]. It has been postulated this inhibition of glucan synthesis in the cell wall results in less β-1,3 glucan in the matrix [35]. We posit that either the production or assembly enzymes of importance for the mature MGCx may be promising therapeutic targets. Attempts to hydrolyze these polysaccharide and nucleic acid matrix components have been successful in sensitizing both *Candida* and *Aspergillus* biofilms to available antifungals [26,27]. The combination of existing antifungals with a hydrolyzing enzyme may be useful for mucosal or topical applications. However, disruption of systemic biofilms, such as those on vascular catheters, would be anticipated to enhance cell dispersion and production of disseminated infection, which would consequently require additional pharmacological interventions both locally for biofilm and systematically to eradicate microorganisms migrating to tissues and organs.

Peptides targeting the host and microbe matrix interactions have also demonstrated efficacy in treatment and prevention studies both in vitro and in vivo [36]. Specifically, targeting of host protein binding to the fibronectin binding site in *C*. *albicans* was modestly effective in inhibiting biofilm formation. The identification and targeting of these additional matrix targets, particularly those that are conserved among pathogens, holds promise for effective prevention and treatment strategies.

The biofilm matrix represents a complex interaction of multiple macromolecular components. A role in cell protection has been identified for several of these constituents. However, the function for the majority of matrix elements remains unexplored. Deciphering the exact composition and roles for these materials should lead to advances in biofilm prevention, therapy, and diagnosis.
